# Vascular dementia: World Stroke Organization fact sheet 2026

**DOI:** 10.1177/17474930251404243

**Published:** 2026-01-01

**Authors:** Yuan Cai, Vincent Chung Tong Mok, Hugh S Markus

**Affiliations:** 1 Lau Tat-chuen Research Centre of Brain Degenerative Diseases in Chinese, Lui Che Woo Institute of Innovative Medicine, Gerald Choa Neuroscience Institute, Li Ka Shing Institute of Health Science, Division of Neurology, Department of Medicine and Therapeutics, Faculty of Medicine, The Chinese University of Hong Kong, Prince of Wales Hospital, Hong Kong SAR, China; 2 Stroke Research Group, Department of Clinical Neurosciences, University of Cambridge, Cambridge, UK

**Keywords:** Vascular cognitive impairment, small vessel disease, Alzheimer’s Disease, stroke, dementia

## Abstract

There were 56.9 million people worldwide living with dementia in 2021, according to the Global Burden of Disease study, and this number is projected to exceed 137 million by 2050. Vascular dementia (VaD) is the second leading cause of dementia. While high-quality global epidemiological data on VaD remain limited, population-based studies with autopsy confirmation allow an approximate estimation. These show that pure VaD represents approximately 15% of all dementia cases, with mixed vascular and degenerative dementia accounting for an additional 16%. According to these estimates, approximately 8.5 million people worldwide suffer from pure VaD, and 9.1 million from mixed dementia. Under the assumption that existing proportional rates remain constant, the global burden of total VaD (i.e. pure VaD and mixed dementia) will reach 42.7 million cases by 2050. However, the impact of cerebrovascular disease is likely to be even greater. Increasing evidence demonstrates that vascular pathology commonly coexists with Alzheimer’s and other neurodegenerative pathologies, increasing the risk that these neurodegenerative pathologies cause clinical dementia. Despite the importance of VaD, it remains underrecognized and underresearched compared to other forms of dementia. This fact sheet highlights the urgent need for improved recognition, standardized diagnostic approaches, and enhanced preventive strategies for this highly prevalent yet underrecognized cause of dementia. The factsheet has been reviewed and approved by the World Stroke Organization (WSO) executive.

## Graphical abstract

**Graphical abstract:**
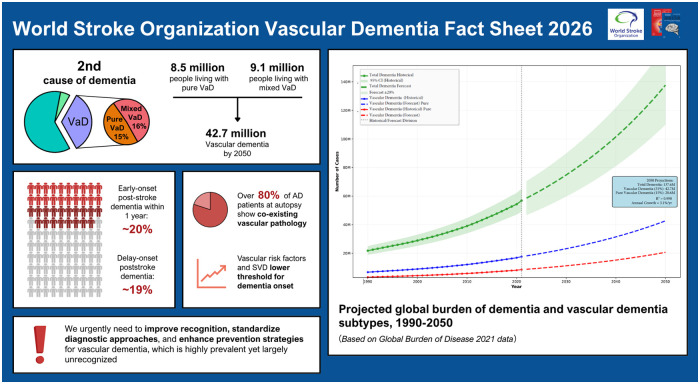


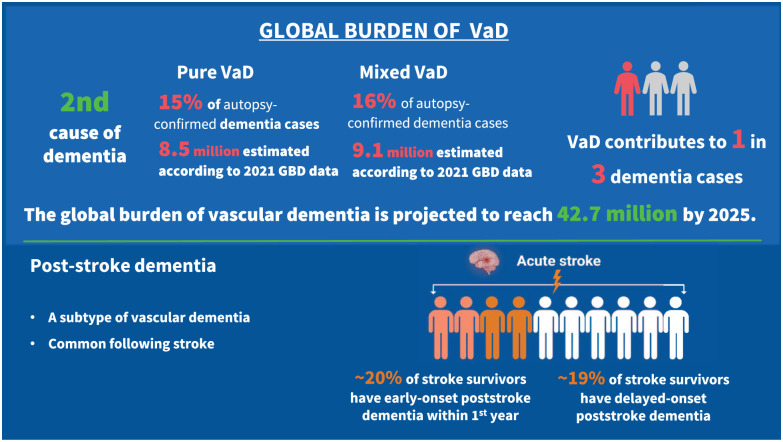


## Introduction

Vascular dementia (VaD) is the second leading cause of dementia. With an increasing global stroke burden, both due to an aging population and increased incidence in low and middle-income countries (LMICs), its burden is expected to increase.^1^ However, it has received less attention than neurodegenerative dementias such as Alzheimer’s, and high-quality global data on VaD prevalence remains limited.

To assess the global burden of VaD, this factsheet presents a number of different analyses. First, it examines epidemiological estimates for both pathologically defined and clinically defined VaD. It highlights challenges in clinical diagnosis, which makes deriving exact prevalence estimates from clinical data challenging. Using these data, it presents epidemiological estimates for VaD where available, assesses the proportion of dementias caused by VaD, and then derives the contribution of VaD to the total burden of dementia. Next, it examines a specific situation, the risk of dementia in individuals who have suffered a stroke, post-stroke cognitive impairment (PSCI), and dementia. Finally, it estimates the additional potential contribution of the vascular contribution to neurodegenerative dementias, an area of increasing research and clinical interest.

## Methods

For epidemiological data obtained from population-based studies, we performed a comprehensive systematic search of PubMed on 15 June 2025, employing keywords such as “vascular dementia,” “mixed dementia,” “population-based,” “pathological diagnosis,” and “clinical diagnosis.” Our search targeted population-based studies that reported prevalence data for VaD and mixed dementia (i.e. dementia caused by vascular pathology combined with other pathological changes) with either neuropathological or clinical-based diagnosis. In addition, an analysis of the data from the UK Biobank was conducted using the UK Biobank Resource (https://www.ukbiobank.ac.uk/) under Application Number 36509. We then systematically extracted data from eligible studies on diagnostic methodologies, case numbers, sample characteristics, and demographic variables. We conducted meta-analyses using random-effects models specifically for studies with neuropathologically confirmed diagnoses.

For the projection of the dementia data prevalence of 2021-2050, data were obtained from the GBD 2021 conducted by the Institute for Health Metrics and Evaluation, extracting global prevalence estimates for dementias of absolute case numbers for 1990–2021. Data are available from https://vizhub.healthdata.org/gbdresults/ and downloaded from the GBD results platform using “Alzheimer’s disease and other dementias” as a Level 4 cause of death or injury, extracting key metrics (number, percent, rate) for measures.

Dementia prevalence projections through 2050 were generated using linear regression on transformed data following the specification log(Yt) = β_0_ + β_1_(t − t_0_) + εt. VaD burden was estimated through proportional allocation based on postmortem diagnosed epidemiological evidence: total VaD (i.e. combined pure VaD and mixed dementia) as 31% of total dementia burden and pure VaD as 15%. Model performance was assessed using the coefficient of determination (R^2^), with historical 95% confidence interval (CI) preserved from GBD 2021 estimates and ±20% symmetric uncertainty bounds applied to projections to account for model uncertainty. All analyses were conducted in Python 3 and R software (version 4.3.3).

## Epidemiology and pathological evidence of VaD

### Epidemiological data based on clinicopathological diagnosis

**Figure 1.**
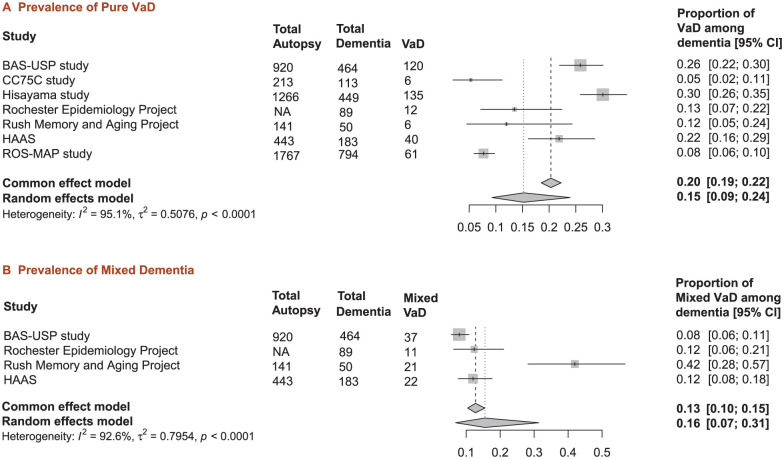
Meta-analysis of prevalence of pure vascular dementia and mixed dementia in population-based autopsy studies. Forest plots showing pooled prevalence from population-based studies. (a) Pure VaD: seven studies, pooled prevalence 15% (95% CI: 9–24%). (b) Mixed dementia: four studies; pooled prevalence 16% (95% CI: 7–31%). For mixed dementia, only the data from Rush Memory and Aging Project included subjects with AD, VaD, and other concurrent pathologies (n = 2), while others reported only mixed AD and VaD pathology. Square symbols: individual studies; diamonds: pooled estimates. High heterogeneity observed in both analyses (I^2^ > 90%). Abbreviation: BAS-USP: Brazilian Biobank for Aging Studies; CC75C: City over−75s Cohort Study; HAAS: Honolulu Asia Ageing Study; ROS−MAP: Rush Religious Orders Study−Memory and Aging Project.

We reviewed seven population-based postmortem studies from diverse geographic regions. The prevalence of pure VaD varied substantially across studies, ranging from 5% in the Cambridge City over-75s Cohort Study(CC75C) to 30% in the Biobank for Aging Studies of the University of Sao Paulo (BAS-USP) study (Figure 1(a)).^2–8^ Mixed dementia prevalence also showed considerable variation, ranging from 8% in BAS-USP study to 42% in the Rush Memory and Aging Project (Figure 1(b)). There is substantial heterogeneity between studies (I^2^ = 95.1% for pure VaD, 92.6% for mixed dementia), reflecting methodological and population differences. In the Rochester Epidemiology Project, Rush Memory and Aging Project, and Honolulu Asia Ageing Study (HAAS), pure VaD was pathologically diagnosed in clinically diagnosed dementia cases where cerebral infarctions were the only significant pathological finding. In the CC75C, dementia subjects with vascular disease as the first rank diagnosis in the neuropathologist’s report were classified as pure VaD. In the Rush Religious Orders Study-Memory and Aging Project (ROS-MAP), pure VaD was defined as dementia subjects with pure cerebrovascular pathologies (i.e., macroinfarcts, microinfarcts, atherosclerosis, arteriolosclerosis) in the absence of significant neurodegenerative brain pathologies. The specific criteria for defining VaD were not explicitly described in the published reports for the Hisayama study and BAS-USP study. Among studies reporting mixed dementia, only the Rush Memory and Aging Project identified subjects with AD, VaD, and other concurrent pathologies (n = 2), while others reported only mixed AD and VaD pathology. Notably, only one study was conducted in a low- to middle-income country (Brazil). A meta-analysis using a random-effects model showed that autopsy-confirmed pure VaD accounted for 15% of clinical diagnosed dementia cases (95% CI: 0.09–0.24, Figure 1(a)), while mixed vascular and degenerative pathology accounted for 16% of cases (95% CI: 0.07–0.31, Figure 1(b)).

### Epidemiological data based on clinical criteria

Based on recent nine population-based studies with sample sizes ranging from 927 to 446,814 participants, the proportion of VaD among all dementia cases varied considerably across studies, from 10.4% in Norway to 46.7% in rural Japan (1997), with intermediate values including 13.9% in Korea, 17.9% in UK biobank, 26.1% in China, and 20.3% in Hong Kong (Table 1).^9–17^ Significant heterogeneity was observed across studies (I^2^ = 95.7%), likely reflecting differences in diagnostic criteria, geographic regions, and study methodologies. Using a random-effects meta-analysis model, the pooled proportion of VaD was 25% (95% CI: 0.18–0.32), suggesting approximately one-quarter of community-dwelling dementia cases can be attributed to vascular etiology

**Table 1.** Prevalence of vascular dementia among selected population-based studies.

Country/Region	Study year	Recruitment Age /Birth Year	Sample size	Prevalence of vascular dementia	Numbers of dementia cases	Propotion of VaD among dementia cases	Vascular dementia diagnosis criteria
Japan (rural area)^16^	1997, 2004, 2012, 2016	65+	1997: 1220. 2004: 1290. 2012: 1129. 2016: 927	Age-standardized: 1997: 2.1%, 2004: 1.8%, 2012: 1.8%, 2016: 2.4% Crude: 1997: 2.3% 2004: 2.1% 2012: 2.8% 2016: 3.3%	1997: 28 2004: 27 2012: 31 2016: 31	1997: 46.7% 2004: 29.3% 2012: 29.8% 2016: 20.5%	DSM-IV (1997, 2004, 2012); NINDS-AIREN (2016)
Iceland-Reykjavik^11^	2002–2006	Birth year 1907–1935	3906	1.3%	132	38.6%	ADDTC
Korea^10^	2009	60+	6481	0.6%	287	13.9%	NINDS-AIREN criteria
Mexico^12^	2012–2015	50+	14,893	0.6%	NA	NA	Self-reported: stroke history + dementia
China^9^	2015–2018	60+	46,011	1.6%	2766	26.1%	NINDS-AIREN criteria
Norway-Nord-Trøndelag County^13^	2017–2019 (HUNT4)	70+	9720	1.6%	1503	10.4%	DSM-V criteria
China (rural area)^14^	2018	60+	5715	1.7% VaD prevalence	306	32.7%	NINDS-AIREN criteria based essentially on the history of stroke
China-Hong Kong	2023	No	6100	20.3% of all-cause dementia cases	NA	NA	per psychiatrists’ clinical judgment
United Kingdom	2024	40+	44,6814	0.5%	11643	17.9%	ICD-10

Available longitudinal cohort studies (n = 9) encompassing 1087 to 248,190 participants with follow-up periods ranging from 1 to 44 years demonstrated significant heterogeneity in VaD incidence rates (Table 2).^12,18–25^ The reported incidence rates show considerable variation across different populations, ranging from approximately 0.08 to 0.33 per 100 person-years. When examining VaD as a proportion of all-cause dementia, rates varied from 9% to 22% across different populations. Mixed dementia accounted for an additional 21–27% of all dementia cases. Combined vascular-related dementia (pure VaD plus mixed dementia) represents approximately 25–50% of all dementia cases across the studies examined.

**Table 2.** Incidence of vascular dementia among selected longitudinal population-based studies.

Country/Region	Study year	Recruitment age	Sample size	Incidence of vascular dementia	Proportion among dementia cases	Follow-up years	Vascular dementia diagnosis criteria
USA^18^	1988–1989	65+	3608	3.8 per 1000 persons by pre-MRI diagnosis	pre-MRI diagnosis classified: 10.8% DSM-IV criteria: 12.7% ADDTC criteria: 44.4% NINDS-AIREN criteria: 11.0%	5.4 years	Multiple diagnostic criteria: Pre-MRI diagnosis; DSM-IV, NINDS-AIREN, ADDTC criteria;
Mexico^12^	2012–2015	50+	12,427	87 new VaD cases, 2.2/1000 person-years	NA	3 years	Self-reported: stroke history + dementia
Finland-Northern Savo area^19^	2020	60+	248,190	202 new VaD cases, annual incidence: 81.4 per 100,000 (overall population), 310.8 per 100,000 (age 65+)	NA	1 year	ICD-10 code
Sweden-Malmö^20^	2002–2009	Mean age 69 ± 6 years	5323	80 new VaD, mean 0.33 per 100 person-years 101 new mixed dementia, mean 0.41 per 100 person-years	VaD: 21.7% Mixed dementia: 27.4%	4.6 years	DSM-IV criteria
Netherlands-Rotterdam^21^	1990–2004	55+	5553	73 new VaD, mean 0.11 per 100 person-years	11.1%	11.6 years	NINDS-AIREN criteria
France^22^	1999–2004	65+	7087	40 new VaD, mean 0.16 per 100 person-year	19.2%	at least 4 years	NINDS-AIREN criteria
USA^23^	1987–2016	45+	4559	436 new vascular MCI + dementia	35.6% of all-cause MCI + dementia	15 years	NINDS-AIREN criteria
Sweden-Gothenburg^24^	1968–2010	38+	1447 women	52 new VaD, mean 0.08 per 100 person-years 66 new mixed dementia cases, mean 0.10 per 100 person-years	VaD: 16.3% Mixed dementia: 20.6%	44 years	NINDS-AIREN criteria based essentially on the history of stroke
USA-Washington^25^	1994–2018	65+	4743	117 new VaD, mean 0.28 per 100 person-years	9.2%	8.7 years	DSM-IV criteria

## Limitations in pathological and clinical prevalence estimates of VaD

There are two major limitations with the above estimates: limitations in the accuracy of clinical diagnostic criteria, and variability in the diagnostic criteria themselves. Multiple studies have examined the diagnostic specificity, sensitivity, and consistency of the currently widely used criteria, including DSM-IV/V, NINDS-AIREN,^26^ and ADDTC criteria.^27^ The application of different diagnostic standards for VaD within the same population can result in diagnostic rates that vary dramatically, ranging from as low as 10% to over 90%.^18,28,29^ In general, ADDTC detects a higher proportion of VaD patients than NINDS-AIREN. There is poor consistency across different diagnostic criteria, indicating that different clinical diagnostic criteria systematically select distinct patient populations that cannot be considered interchangeable.

Although autopsy studies can help us better identify the causes of dementia and classify it, there are currently no comprehensive, unified standards for the diagnosis of vascular pathology.^30^ A large pooled analysis based on six population studies found that when different pathologies were harmonized across studies, cerebrovascular pathology (arteriosclerosis, atherosclerosis, cerebral amyloid angiopathy, and lacunar infarction) showed low reliability during the standardization process.^31^ Despite the proposed Vascular Cognitive Impairment Neuropathology Guidelines (VCING),^32^ the field lacks rigorously validated pathological diagnostic criteria that have been subjected to systematic clinical investigation. In addition, the clinical-pathological diagnostic discordance further limits VaD estimates. The International Statistical Classification of Diseases and Related Health Problems, 10th Revision (ICD-10) diagnostic criteria exemplify this problematic discordance. While ICD-10 demonstrates good interrater agreement and widespread adoption in multiple cohort studies,^33,34^ its sensitivity against neuropathological standards is only 20%.^[Bibr bibr34-17474930251404243] 35^ This indicates the majority of true VaD cases may remain undiagnosed, potentially compromising the generalizability and validity of genetic and epidemiological findings. Although ADDTC and NINDAREN demonstrated a better balance between specificity and sensitivity, they only achieved approximately 50% sensitivity and 70% specificity.^30,36^

These diagnostic limitations across both clinical criteria and pathological standards limit the reliability of VaD epidemiological data and research findings. The field urgently requires standardized, validated diagnostic frameworks that bridge the clinical-pathological divide to ensure accurate disease characterization and meaningful research outcomes.

## PSCI and dementia

**Figure 2.**
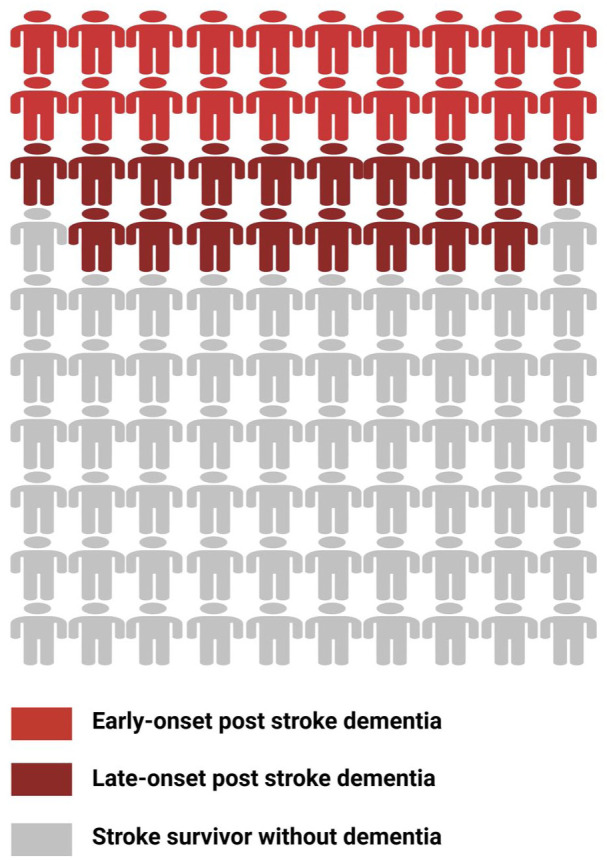
Schematic diagram of the risk of dementia and cognitive impairment after stroke. Visual representation of the risk of cognitive impairment and dementia in stroke survivors, derived from a summary of previous published studies. Candy red figures represent patients with early-onset post-stroke dementia within 1 year after stroke, affecting approximately 10–20% of stroke survivors. In addition, nearly 40% of patients developed non-dementia PSCI. Dark red figures represent patients without early-onset dementia but who developed delayed-onset post-stroke dementia, affecting approximately 19% of stroke survivors. Gray figures represent stroke survivors without dementia.

Stroke commonly leads to cognitive decline after the stroke event (Figure 2). Depending on the study population and the definition of outcome events, systematic reviews show the prevalence of early-onset post-stroke dementia (PSD) within 1 year after stroke ranged from 7.4% in population-based studies of first-ever stroke without pre-stroke dementia, to 41.3% in hospital-based studies of recurrent stroke in which pre-stroke dementia was included. In hospital-based studies without pre-stroke dementia, the pooled prevalence of dementia is approximately 20.3%.^37^ Meanwhile, a previous meta-analysis suggests that 38% of stroke patients may develop cognitive impairment without dementia in the first year post-stroke.^38^ A combined analysis of 3146 participants without pre-stroke dementia also found a high prevalence of early-onset PSCI, with 44% developing global cognitive impairments 2 to 6 months after stroke.^39^ Stroke severity is a strong predictor of the incidence of early-onset PSCI. In the population-based longitudinal Oxford Vascular Study, the 1-year incidence of PSD was 5.2% (95% CI: 3.4–7.0) for transient ischemic attack (TIA), 8.2% (6.2–10.2) for minor stroke, and 34.4% (29.7–41.5) for severe stroke. Compared with age-matched controls, this represented standardized morbidity ratios of 3.5-, 5.8-, and 47.3-fold increases, respectively, demonstrating that severe stroke confers an almost 50-fold higher risk of developing dementia within the first year.^40^

Survivors who do not experience cognitive decline in the early stages of stroke may also develop late-onset PSCI, and tend to exhibit faster cognitive decline after stroke/TIA than healthy non-stroke subjects of the same age.^41,42^ A large population-based study using self-reported stroke diagnoses found that among first-time stroke patients, pre-stroke cognitive decline rates were identical to those of healthy individuals, but post-stroke cognitive decline accelerated to approximately 1.8 times the normal age-related cognitive decline rate observed in healthy older adults.^43^ Similarly, data from the Framingham Study shows that the risk of developing dementia 10 years after a stroke is 19.3%, compared to 11.0% for those without a stroke.^44^ Meanwhile, it has also been reported that incident dementia risk almost doubled among hospitalized TIA subjects compared to those without TIA during 29 years of follow-up.^45^ This delayed presentation poses diagnostic challenges, as the absence of clear temporal continuity with stroke onset often leads to misclassification under the broader umbrella term of dementia, obscuring its identification as vascular cognitive impairment. The development of delayed-onset PSCI is influenced by several factors, including the presence of chronic cerebral small vessel disease (SVD) imaging changes such as white matter hyperintensities and lacunes.^41,46^ Notably, risk factors for early-onset and late-onset PSD may differ. A recent prospective multicenter cohort study demonstrated that stroke severity was more strongly associated with early-onset PSD, while metabolic syndrome showed a greater contribution to late-onset PSD.^47^

## A global estimate of VaD prevalence and future trends

**Figure 3.**
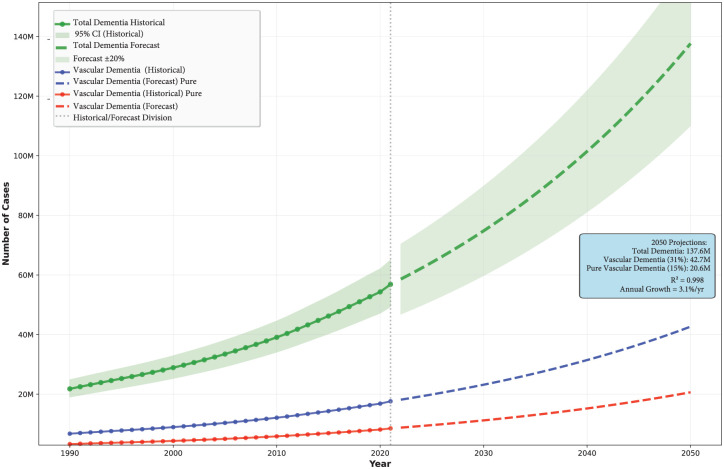
Projected global burden of dementia and vascular dementia subtypes, 1990–2050. Historical trends (solid lines, 1990–2020) and future projections (dashed lines, 2020–2050) for total dementia cases (green), vascular dementia cases assuming 31% prevalence among all dementia (blue), and pure vascular dementia cases assuming 15% prevalence (red). The vertical dotted line indicates the transition from historical data to forecast projections. Shaded areas represent 95% confidence intervals for historical estimates (light green) and ±20% forecast uncertainty bounds (light green). Projections indicate total dementia cases reaching 137.6 million by 2050, with vascular dementia (31% assumption) accounting for 42.7 million cases and pure vascular dementia (15% assumption) for 20.6 million cases.

According to the Global Burden of Disease (GBD 2021) study, there are 56.9 (95% CI: 49.4–65.0) million people worldwide living with dementia in 2021, and this number is projected to be 137.6 million by 2050 (Figure 3).^48^ Based on the above analyses, pure VaD represents approximately 15% of total dementia cases, with mixed vascular and degenerative dementia accounting for an additional 16% (Figure 1). According to these estimates, approximately 8.5 million people worldwide suffer from pure VaD, and 9.1 million suffer from mixed dementia in 2021. Under the hypothetical assumption that existing proportional rates remain constant, the global burden of total VaD (i.e. pure VaD and mixed dementia) may potentially reach 42.7 million cases by 2050 (Figure 3).

**Figure 4.**
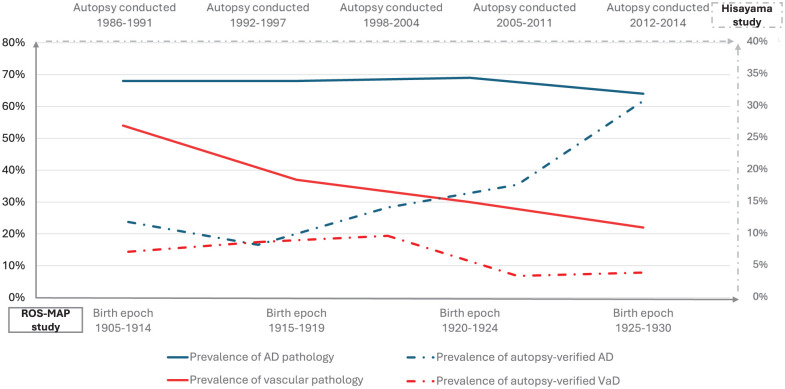
Trends in prevalence of AD and vascular pathology in ROS-MAP and Hisayama study. Data from the Religious Orders Study and Rush Memory and Aging Project (ROS-MAP, birth epoch 1905–1930, the bottom x-axis) and the Hisayama Study (autopsy date 1986–2014, the top x-axis) show prevalence rates across different time points. The left y-axis represents prevalence percentages for pathological findings in ROS-MAP (0–80%), while the right y-axis represents prevalence percentages for autopsy-verified clinical diagnoses in Hisayama Study (0–40%). Solid line: prevalence of AD/Vascular pathology in ROS-MAP; Dashed line: prevalence of autopsy-verified AD/VaD diagnosis in Hisayama Study. Both studies demonstrate a consistent declining trend in vascular pathology and autopsy-confirmed vascular dementia, while AD pathology prevalence remained relatively stable and autopsy-verified AD diagnosis showed an increasing trend.

Future trends in VaD prevalence are difficult to accurately predict. Population-based studies suggest that while the incidence of stroke may be decreasing in high-income countries (HICs), this decrease may have recently plateaued,^49^ and the incidence is increasing in LMICs.^1^ Longitudinal data on changes in vascular pathology prevalence remain limited. However, findings from the ROS-MAP study^50^ and the Hisayama Study^4^ revealed a strikingly similar trend: both showed a declining prevalence of vascular pathology and autopsy-confirmed VaD in the general population, while Alzheimer’s disease pathology remained relatively stable or even showed an upward trend (Figure 4). These studies are from higher-income countries and may parallel the reported reduction in stroke incidence reported in HICs. They do not necessarily reflect the picture in LMICs, and therefore, the future global burden of VaD in LMICs. However, the reduction seen in stroke incidence in HICs, and the corresponding reduction in overall dementia incidence in these countries, suggests that preventive and management strategies over recent decades may have been effective. The 2024 Lancet Commission identified 14 modifiable risk factors that together could reduce overall dementia risk by 45%, including hypertension, diabetes, smoking, physical inactivity, obesity, and hyperlipidemia.^51^

## The impact of cerebrovascular burden on neurodegenerative dementias

**Figure 5.**
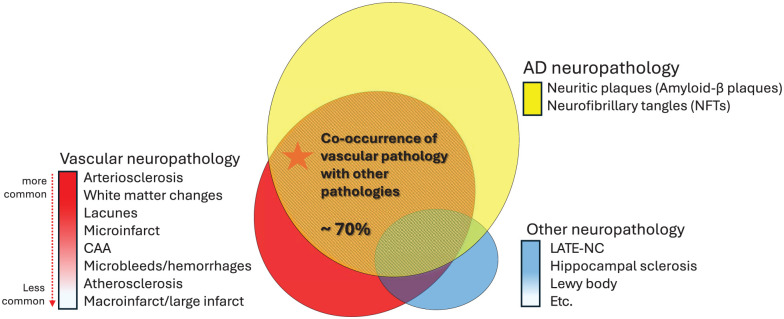
Co-occurrence of vascular and other neuropathologies in dementia. Venn diagram showing overlapping neuropathologies: AD pathology (yellow), vascular pathology (red), and other pathologies (blue). Central overlap shows ~70% co-occurrence rate of vascular pathology with other neuropathologies. Vascular pathologies are arranged from more common (arteriosclerosis, white matter changes) to less common (large infarcts) manifestations.

The above estimates include both pure VaD and mixed dementias, but do not account for a possible role of vascular risk in accelerating neurodegenerative dementias. Autopsy studies in general populations demonstrate that co-existence of vascular pathology and degenerative changes is common, and is in fact the most common picture (Figure 5).^31^ Multiple studies have demonstrated that 48–80% of AD patients at autopsy show coexisting vascular pathology.^52–54^ Similarly, in autopsy-confirmed frontotemporal dementia (FTD) and α-synucleinopathies, over 60% of patients also presented with vascular pathology.^8,54^ Among these, cerebral amyloid angiopathy (CAA) has been reported with high prevalence rates in AD patients.^55^ Imaging or pathological appearances of SVD appears to be also a highly prevalent and vital modifying pathology,^31,56^ and have been shown to markedly lower the threshold at which AD pathology identified postmortem results in clinical dementia during life.^57^ Increasing evidence suggests that vascular risk factors and SVD may also have a direct effect on accelerating AD pathology, perhaps via pathways such as impairing the clearance of amyloid β and tau protein.^58^ Therefore, reducing the cerebrovascular burden has the potential to have a significant impact on reducing the burden of all dementias.

## Summary

The summary of the global VaD burden is presented in key points table. The evidence presented demonstrates that vascular dementia represents a substantial and escalating global burden, placing significant strain on healthcare systems worldwide. As the global population ages, addressing vascular contributions to cognitive decline will be essential for reducing the overall burden of dementia. Enhanced focus on vascular dementia research, improved diagnostic tools, and prevention strategies represents a critical pathway toward more effective dementia care and prevention.

## Vascular dementia (VaD)—key points

**Table:** 

	Key points
Epidemiology	• 2nd leading cause of dementia • Includes pure VaD and mixed dementia, up to 31% of all dementia cases • ~17.6 million people affected globally • Projected to reach 42 million by 2050
Post-Stroke Cognitive Impairments	• Up to 44% of stroke patients develop cognitive impairment • Severe stroke increases dementia risk almost 50-fold • 10 year post-stroke dementia risk: 19.3%
Diagnostic Challenges	• Clinical diagnosis sensitivity is only 20-50% • Poor consistency among different diagnostic criteria has led to significant research heterogeneity. • Lack of unified diagnostic criteria • Often coexists with other pathologies
Relationship with Other Pathologies	• 48–80% of AD patients have coexisting vascular pathology • Vascular factors increase risk of Alzheimer’s disease • Lowers threshold for dementia onset
Clinical Significance	• Highly preventable • Under-recognized and under-diagnosed • Requires multidisciplinary management
